# Development and characterization of a continuous cell line (EL) from the liver of European eel *Anguilla anguilla*

**DOI:** 10.1002/cbin.11276

**Published:** 2019-12-19

**Authors:** Zaiyu Zheng, Jinxian Yang, Junqing Ge, Hongshu Chi, Bin Chen, Qinmei Fang, Hui Gong

**Affiliations:** ^1^ Biotechnology Institute Fujian Academy of Agricultural Sciences Wusi Road 247 Fuzhou Fujian 350003 China; ^2^ Ningde Fufa Fisheries Company Ltd. Ningde Fujian China

**Keywords:** *Anguilla anguilla*, hepatic cell line, *Herpesvirus anguillae*, immune‐related gene, *Rana grylio* virus

## Abstract

In the present study, a new hepatic tissue‐origin cell line from European eel *Anguilla anguilla* has been developed and characterized. This cell line designated EL has been maintained in Leibovitz L‐15 supplemented with 10% fetal bovine serum over 72 months, and subcultured more than 90 times. The EL cell line consisted predominantly of fibroblast‐like cells, which could survive over 100 days in vitro, and could grow at 15–32°C. The optimum temperature for growth was 27°C. The chromosome analysis revealed a modal diploid karyotype of 2*n* = 38. The origin of this cell line was confirmed by the 18S recombinant (r)RNA sequencing. The susceptibility test indicated significant cytopathic effects in the EL cells with regard to the *Rana grylio* virus and the *Herpesvirus anguillae*. The viral replication was confirmed by transmission electron microscopy and polymerase chain reaction analysis. Following poly (I:C) exposure, the expression levels of the immune‐related molecules interferon regulatory factor‐7 (*irf7*) and transforming growth factor‐*β* (TGF‐*β*) were downregulated in EL cells, whereas the expression levels of the *rf3* and the cytochrome P450 (*CYP450*) were upregulated. All four genes were significantly upregulated following inflammation by lipopolysaccharide (*LPS*). These data suggested the application of EL cell line for viral identification, as well as for immunodiagnosis and pharmacological targeting.

**Abbreviations**
*irf3*: interferon regulatory factor‐3
*irf7*: interferon regulatory factor‐7
CPE: cytopathic effects
CYP450: cytochrome P450
dNTP: deoxynucleotide triphosphate
FBS: fetal bovine serum
HVA: *Herpesvirus anguillae*
MCP: main capsid protein
PDT: population doubling time
RGV: *Rana grylio* virus
TGF‐β: transforming growth factor β
VF: viral factories

## Introduction

In the studies of aquatic virology and toxicology, fish cell lines are considered classical models and viable tools in the fields of immunology, pharmacology, and genetics (Baksi and Frazier, 1990; Kohlpoth et al., 1999; Ni Shúilleabháina et al., 2006). From the early 1960s, almost 400 fish cell lines have been established (Fryer and Lannan, 1994; Lakra et al., 2011), and used for the investigation of aquatic diseases as well as for environmental monitoring (Béjar et al., 2002; Zhang et al., 2003; Chen et al., 2005; Bryson et al., 2006; Sahul‐Hameed et al., 2006; Dong et al., 2008).

The European eel *Anguilla anguilla* (L. 1758) is a fish species that has been traditionally used as a main food‐consumption product in Europe (Dekker, 2018). *A. anguilla* was first introduced into China in 1993. In the late 1990s’ and in the first decade of the present century, *A. anguilla* became the most productive aquacultured Anguillidae species in China. The failed artificial breeding of eels rendered the whole eel industry capture‐based, relying on the capture of wild juvenile glass eels (Politis et al., 2017). In the last 50 years, the abundance of *A. anguilla* in its original habitat has decreased over 90% (Dekker, 2004, 2018), In 2009, *A. anguilla* was listed CR (critically endangered) by International Union for Conservation of Nature (IUCN), while the precious species is still threatened by various pathogens and pollutants (Yue et al., 1998; Zhang and Gui, 2007; Jakob et al., 2009; Hendriks et al., 2010; Galinier et al., 2012; Ge et al., 2012; van Beurden et al., 2012; Fichtner et al., 2013). The disease prevention and protection of *A. anguilla* requires additional viable in vitro cell line tools. To date, the Anguillidae invitrome remains considerably limited (Table 1) (Bols et al., 2017). Several cell lines have been derived from *Anguilla japonica* (Temminck and Schlegel 1846) in the last century (Chen and Kou, 1981; Chen et al., 1982; Kou et al., 1995), while only two cell lines were reported to originate from *Anguilla rostrata* (Lesueur 1817) (Dewitte‐Orr et al., 2006; Bloch et al., 2016) in recent years. However, a limited number of cell lines have been developed from *A. anguilla*. Our lab attempted for the first time the in vitro tissue and cell culture of multiple European eel organs in 2007 (Zheng, 2008) and the first viscera cell line of *A. anguilla* has been established (Chen et al., 2019).

**Table 1:** **The Anguillidae origin cell lines**. Six Auguillidae origin cell lines established within the last four decades are summarized in this table: the names and characteristics of these cell lines and references to the original description are available

No.	Designation	Species	Tissue	Morphology	Incubator	Characterization	References
1.	EO‐2	*Anguilla japonica*	Ovary	Fibroblastic	32℃	Sensitive to EVE, EVEX, and EVA	Chen and Kou (1981)
2.	EK‐1	*Anguilla japonica*	Kidney	Epithelial‐like	26℃	In vitro cultivation model for fish microsporidia	Chen et al. (1982)
3.	EP‐1	*Anguilla japonica*	Viscera	Squamous and fibroblast‐like	28℃	Persistently infected with *Plesitophora anguillarum*	Kou et al. (1995)
4.	PBLE	*Anguilla rostrata*	Leukocytes	Fibroblastic	18℃	Susceptible to CSRV	Dewitte‐Orr et al. (2006)
5.	eelB	*Anguilla rostrata*	Brain	Endothelial‐like	18℃	Express vWF and claudin‐5	Bloch et al. (2016)
6.	EK	*Anguilla anguilla*	Kidney	Fibroblast‐like	26℃	Susceptible to RGV	Chen et al. (2019)

To date, no cell line has been produced from Anguillidae hepatic tissues. The hepatic‐origin cell lines (Fryer et al., 1981; Lannan et al., 1984) are usually applied in pharmacological and toxicological studies, such as those that investigate interferon activity assays (Graham and Secombes, 1990), studies that examine the detection of direct‐acting toxins (Babich et al., 1989), and those that investigate the acute exposure to heavy metals and their corresponding intoxicating effects (Misra et al., 1989). In the present study, we developed and characterized a new cell line from the *A. anguilla* liver, which presented a significant longevity and proved to be susceptible to *Rana grylio* virus (RGV) and *Herpesvirus anguillae* (HVA). The genetic responses of EL cells to regular immune stimulations were investigated.

## Materials and methods

### Ethics statement

The present study was approved by the Laboratory Animal Bioethics Committee of the Biotechnology Institute under the approval number *BI‐AEC‐0007055002*. All the experiments were carried out according to the “*Declaration of Helsinki*” (2008) and the Chinese version of “*Guide for the care and use of laboratory animals*” (8th edition; NIH).

### Preparation

Healthy *A. anguilla* elvers with an average weight of 20 g were purchased from an eel farm in Fujian (China). The elvers were monitored in freshwater at room temperature (25–28°C) for 2 weeks in order to confirm their vital status. Prior to the dissection, the fishes were sacrificed by anesthesia using 80 mg/L of MS‐222 (Cantin), and then disinfected by 2% iodine tincture and 75% alcohol three times, respectively, in turn. Sterilized Ca^++^/Mg^++^‐free Dulbecco's Hanks balanced salt solution (D‐Hanks) containing 200 IU/mL of penicillin and 200 μg/mL of streptomycin (Sangon) was prepared and stored at 0°C.

### Primary cell culture and subculture

Fresh hepatic tissues were removed from the eels, washed five times with cold D‐Hanks, and cut into 1 mm^3^ pieces. The inner face of the culture flask was coated with a few drops of serum‐free Leibovitz L‐15 (Hyclone) medium. The tissue fragments were rinsed three times with ice‐cold D‐Hanks and attached to the bottom of 25 cm^2^ culture flasks (Corning). The primary culture was carried out at 20°C and 2 mL of L‐15 containing 15% fetal bovine serum (FBS; Gibco) was added in the presence of antibiotics. Following soaking of explants in the culture medium, 1 mL of additional culture medium was added every 24 h, to increase the total volume to 5 mL. Half of the medium was changed every 72 h, if necessary.

The culture was digested with 0.25% trypsin solution (Sigma‐Aldrich) at 27°C for 15 min. Following centrifugation, the cells and tissue residues were collected and suspended in 5 mL complete culture medium that supplemented with FBS and antibiotics. The cells were cultivated in new 12.5 cm^2^ flasks (BD falcon) at 27°C. The multiplication ratio of the first several subcultures was retained from 2:1 to 1:1 (Wolf and Quimby, 1976).

### Cell line development and storage

A special asymmetric subculture method was set up for the development of the EL cell line. When a confluent cell layer was observed, the subcultures were washed and digested with 0.25% trypsin for 1 min. Subsequently, the supernatants were collected and incubated in a new 25 cm^2^ flask, while the remaining bottom cells were cultured in the original flask unceasingly. From the sixth subculture, the antibiotics were not added to the culture medium. The concentration of FBS in the subculture medium was reduced to 10% at the eighth subculture. From the 60th subculture, the multiplication ratio could be increased to 1:3 (Lannan, 1994).

Following 72–96 h of in vitro growth, the hepatic cells were harvested and re‐suspended in L‐15 containing 20% FBS and 10% dimethyl sulphoxide (DMSO; Sigma‐Aldrich) at a density of 10^6^ cells/mL. The cell suspensions were transferred to 1.2 mL cryogenic vials (Corning), stored initially at 4°C for 30 min and subsequently at −75°C overnight The suspensions were finally stored in liquid nitrogen for long‐term use (−196°C). Following 30 days of cryopreservation, the frozen cells were warmed in a 37°C water bath and subsequently incubated in 25 cm^2^ cell culture flasks. A full medium change was carried 12 h later. The cell viability was evaluated by the Tryphan blue staining method (Ott, 2001).

### Growth studies

Three types of commercial culture medium were used in order to test the optimal medium for EL cell culture. These were Leibovitz L‐15, M199/EBSS, and RPMI‐1640 (Hyclone). The latter two media were used only once. Leibovitz L‐15 was selected and used in the presence of 10% FBS in EL cells at the 6th passage. The flasks were incubated at 27°C and observed daily with a Nikon phase‐contrast microscope.

The growth curve of EL cells was initially plotted until passage number 28, at 27°C. The sampling interval was 48 h. At passage number 66, the EL cells were incubated in 25 cm^2^ flasks (2 × 10^5^ cells per flask) at 15, 20, 27, and 32°C, respectively. Every 24 h, three flasks from each group were harvested and their average cell densities were counted by a hemocytometer. The cell population doubling time (PDT) was determined by the following formula (Davis, 2001):

$$T=t[\;\mathrm{lg}\;2{\left(\mathrm{lg}\;N_{t}\;–\;\;\mathrm{lg}\;N_{0}\right)}^{-1}]$$

### Chromosome analysis

Chromosomal preparation from EL cells was carried out as described previously with some modifications (Chen et al., 2005). Passage 65 EL cells were grown for 36 h and subsequently incubated in L‐15 containing 2% FBS and 1 μg/mL colchicine (Sigma‐Aldrich) at 27°C for 6 h. The cells were harvested and treated with 5 mL of 0.3% KCl for 25 min. For pre‐mixing, 1 mL of Carnoy's fixative (methanol:acetic acid = 3:1, 0°C) was placed gently into the suspension for 5 min. Following centrifugation, the cell pellets were fixed with Carnoy's fixative for 10 min, and the fixed cells were re‐suspended overnight in 1 mL Carnoy's fixative at 4°C. The suspension was placed on cold glass slides, which were air‐dried and stained with 10% Giemsa (pH = 6.8) for 1 h. Under a Nikon Eclipse TE2000‐S fluorescence microscope, 100 metaphase cells were observed and photographed, and their karyotypes were analyzed according to previously published methodologies (Levan et al., 1964).

### 18S rRNA sequence analysis

The authentication of the origin of the EL cell line was performed by sequencing of the 18S recombinant (r) RNA gene (Englisch and Koenemann, 2001; Frankowski and Bastrop, 2010). A pair of specific primers (Table 2) was designed according to the published *A. anguilla* 18S rRNA sequence (GenBank accession no. FM946070.1). Total genomic DNA of passage 66 EL cells was extracted with a GeneJET Genomic DNA Purification Kit (Thermo Fisher Scientific), and amplification was conducted using the polymerase chain reaction (PCR) in a 50 μL reaction mix containing 5.0 μL of 10× buffer, 4.0 μL of deoxynucleotide triphosphate (dNTP) mix (2.5 mM each), 2.0 μL of each primer (10 μM each), 0.5 μL of EX‐taq DNA Polymerase (5 U/μL; Takara) and 2.0 μL of the extracted genomic DNA. The optimal conditions for PCR were the following: Initial denaturation was performed at 94°C for 5 min and 30 cycles were conducted that included denaturation at 94°C for 30 s, annealing at 55°C for 1.5 min, and extension at 72°C for 1 min. A final elongation was performed at 72°C for 10 min. The PCR products (5 μL per sample) were collected with the high pure PCR product purification kit (Roche) and analyzed by electrophoresis in a 1% agarose gel containing 0.5 mg/mL ethidium bromide. The samples were photographed using the FR‐980A gel imaging analysis system (FURI). The DNA samples were recovered and purified with a DNA gel extraction kit (Sangon) and the target DNA fraction was linked to the pMD‐19T vector (Takara) and transfected to the competent *Escherichia coli* strain DH5α. The DNA from the transgenic strain was sequenced by Shanghai Sangon Biological Engineering Technology & Services Co., Ltd.

**Table 2:** **Sequences of polymerase chain reaction (PCR) primers used in the present study**. The sequences of all PCR primers are listed in this table: the 18S rRNA primers were used to prove the origin of the EL cells; the *HVAF/R* and *HVApolF*/*polR* primers were used for HVA detection; the *Ranavirus‐MCP* and *MCP*153F/215R primers were designed for RGV‐MCP quantitative analysis; and the *CYP450*, TGF‐*β, irf3, irf7*, and *β*‐actin primers were applied to measure the expression changes of these immune‐related genes in EL cells after poly(I:C) or *LPS* exposure

Primer name	Forward sequence (5′‐3′)	Reverse sequence (5′‐3′)
*18s* rRNA	TATGCTTGTCTCAAAGATTAAGCCATGC	CACCTACGGAAACCTTGTTACGA
*HVAF/R*	TTGAGGTTGTTGTCGTGCC	CTCTCATGTCATCCAGACGG
*HVApolF*/*polR*	GTGTCGGGCCTTTGTGGTGA	CATGCCGGGAGTCTTTTTGAT
*Ranavirus‐MCP*	AGGCCGACGGTCATGTAG	TTTGTCAAGGAGCACTACCC
*MCP*153F/215R	TCACCAAGCTGCCGTCTCT	AAAACTGCTGCCCGAAAGC
*CYP450*	CTGAGGAAGCCCCAGTACACCA	GGTGCGGACTTTCTTCCAGAG
TGF‐*β*	GCATGGGCTCCTGCACCTA	CGGGGTTGTGATGCTTATAGAG
*irf3*	CCTCAAGAGGTCAGCAAACAAGAA	GCCACCCAATGGAAAAGAAGAG
*irf7*	CGAAGATGCCTATGCCACAGAC	GAGAGTCAAGCCATCCATGTGAT
*β*‐Actin	CCCTTGACTTTGAGCAGGAAATG	CCAGGAAAGAGGGCTGGAACA

### Quantitative real‐time PCR (qRT‐PCR) for RGV detection

The RGV detection test was conducted according to the previous studies (Chen et al., 2019). The primers specific for the RGV major capsid protein (*MCP*) gene amplification and gene detection were synthesized by Shanghai Sangon Biological Engineering Technology & Services Co., Ltd. (http://www.sangon.com) (Table 2). The total DNA of the positive control cells was isolated using GeneJET Genomic DNA Purification Kit (Thermo Fisher Scientific). The *MCP* gene was 695 bp in length and was amplified with a 50 μL PCR reaction mix containing 5.0 μL of 10× *Taq* buffer (20 mM Mg^++^ included), 4.0 μL of dNTP mix (2.5 mM each), 2 μL of each primer, 2 μL of DNA templates, 0.5 μL of Ex *Taq* (5 U/μL^−1^; Takara), and ddH_2_O. The cycling conditions were the following: 95°C for 3 min for initial denaturation, followed by 30 cycles of 94°C for 30 s, 55°C for 30 s, and 72°C for 35 s. The PCR products were collected and purified with a 2% agarose gel and the SanPrep Column DNA Gel Extraction Kit (Sangon).

The MCP‐pMD‐19T ligation was prepared with a pMD‐19T vector Cloning kit (Takara). The new vector was expressed in the *E. coli* DH5*α* strain. The concentration of the amplified plasmid was measured with a NanoDrop 2000 spectrophotometer (Thermo Fisher Scientific) and converted to the corresponding copy number derived from the amplification. The plasmid sample was diluted to 10^10^–10^1^ copies/μL for the construction of a standard curve. The qRT‐PCR was carried out with an Applied Biosystems® 7500 Real‐Time PCR System (Thermo Fisher Scientific) using a SYBR®Premix Ex Taq™ II kit (Takara). The standard amplification reaction was performed under the following conditions: 95°C for 30 s, followed by 40 cycles of 95°C for 50 s and 60°C for 34 s. The dissociation curve determination conditions were the following: 95°C for 15 s, 60°C for 60 s, and 95°C for 15 s.

### Susceptibility test

Two aquatic viruses namely, RGV and HVA were used for EL cell infection to determine its susceptibility. The purified viral samples were prepared according to the previous studies (Liu et al., 2012; Ge et al., 2014). The passage 62 EL cells were incubated at 27°C for 36 h and transferred into the maintenance culture medium (L‐15 containing 2% FBS) 12 h prior to the infection. The supernatants of the infected EO or EPC cells of the two types of viruses were added to the EL cells (100 μL per flask), respectively, and the infected cells were observed daily under a Nikon ECLIPSE TE2000‐S fluorescence microscope to assess the cytopathic effect (CPE). The viability of the virus infected cells was determined the Tryphan blue staining method.

### Electron microscopy

The infected EL cells were pre‐fixed with 2.5% glutaraldehyde in cacodylate buffer (0.1 M, pH 7.4) for 1 min. The cells were subsequently harvested, fixed with 2.5% glutaraldehyde for 2 h at room temperature (25–28°C), and finally incubated overnight at 4°C. Ultra‐thin sections were then prepared, stained, and visualized by Wuhan Goodbio Technology Co., Ltd. (http://www.servicebio.cn).

### PCR detection

Total DNA was extracted from the HVA‐infected cells with a GeneJET Genomic DNA Purification Kit (Thermo Fisher Scientific) at 96 h after infection. The DNA samples were used as templates for PCR identification with two pairs of specific primers, namely *HVAF/R* and *HVApolF/polR* (Table 2). The PCR amplification was conducted according to previously published studies (Shih, 2004; Ge et al., 2012). The PCR products were sequenced and blasted with the published genome sequence of the anguillid herpesvirus 1 strain HVA980811 (Genbank: KX027736.1).

Total DNA was extracted from the RGV‐infected cells at 0, 6, 12, 24, 36, 48, and 72 h following infection with a GeneJET Genomic DNA Purification Kit, and the samples were used as the templates for qRT‐PCR. The viral copy number was calculated with the CT standard curve.

### Induction of immune‐related gene expression

The changes in the expression levels of the interferon regulatory factor‐7 *(irf7*, GenBank accession no. KF577784.1), the interferon regulatory factor‐3 *(irf3*, GenBank accession no. KF577783.1), the transforming growth factor‐*β1 (TGF‐β1*, GenBank accession no. AJ318934.1), and the cytochrome P450 *(CYP450*, GenBank accession no. KF990052.1) genes in the EL cells that were induced by poly (I:C) or lipopolysaccharide (*LPS*) were detected using qRT‐PCR according to a previous study (Wang et al., 2014). Poly (I:C) (final concentration = 10 μg/mL; Sigma) or LPS (final concentration = 1 μg/mL; Sigma) were added to the culture medium, respectively, with an untreated group as the parallel control. The cells were digested and collected following 3, 6, 12, and 24 h of incubation. Total RNA was extracted with a TRIzol® Plus RNA Purification Kit (Invitrogen) and reverse‐transcribed into first‐strand complementary DNA (cDNA) that was used as the template for qRT‐PCR. Reverse transcription was performed with the SuperScript™ III First‐Strand Synthesis SuperMix (Invitrogen). The primers were designed by Primer Premier 6.0 and Beacon designer 7.8 (Table 2) and qRT‐PCR was performed in the CFX384 Touch™ real‐time PCR detection system (Biorad) using the Power SYBR® Green PCR Master Mix reagent (Applied Biosystems). The reaction volume was 20 μL and contained 8.0 μL of ddH_2_O, 10.0 μL of Power SYBR® Green PCR Master Mix, 0.5 μL of each primer and 1.0 μL of the first‐strand cDNA. The cycling conditions were the following: initial denaturation temperature at 95°C for 1 min, followed by 40 cycles of denaturation at 95°C for 15 s, and annealing at 63°C for 25 s. All the gene expression levels were normalized with the internal control gene *β‐actin*, and the $2^{-\Delta \Delta C_{t}}$ method was chosen for the relative expression levels analysis.

### Statistical analysis

Each experiment was repeated at least three times. The data were shown as mean ± standard error (SE) and the statistical significance was determined by one‐way analysis of variance (Turkey's HSD test) (Davis, 2001) using IBM SPSS (http://www.ibm.com/analytics/).

## Results

### Primary cell culture and cell line development

**Figure 1:**
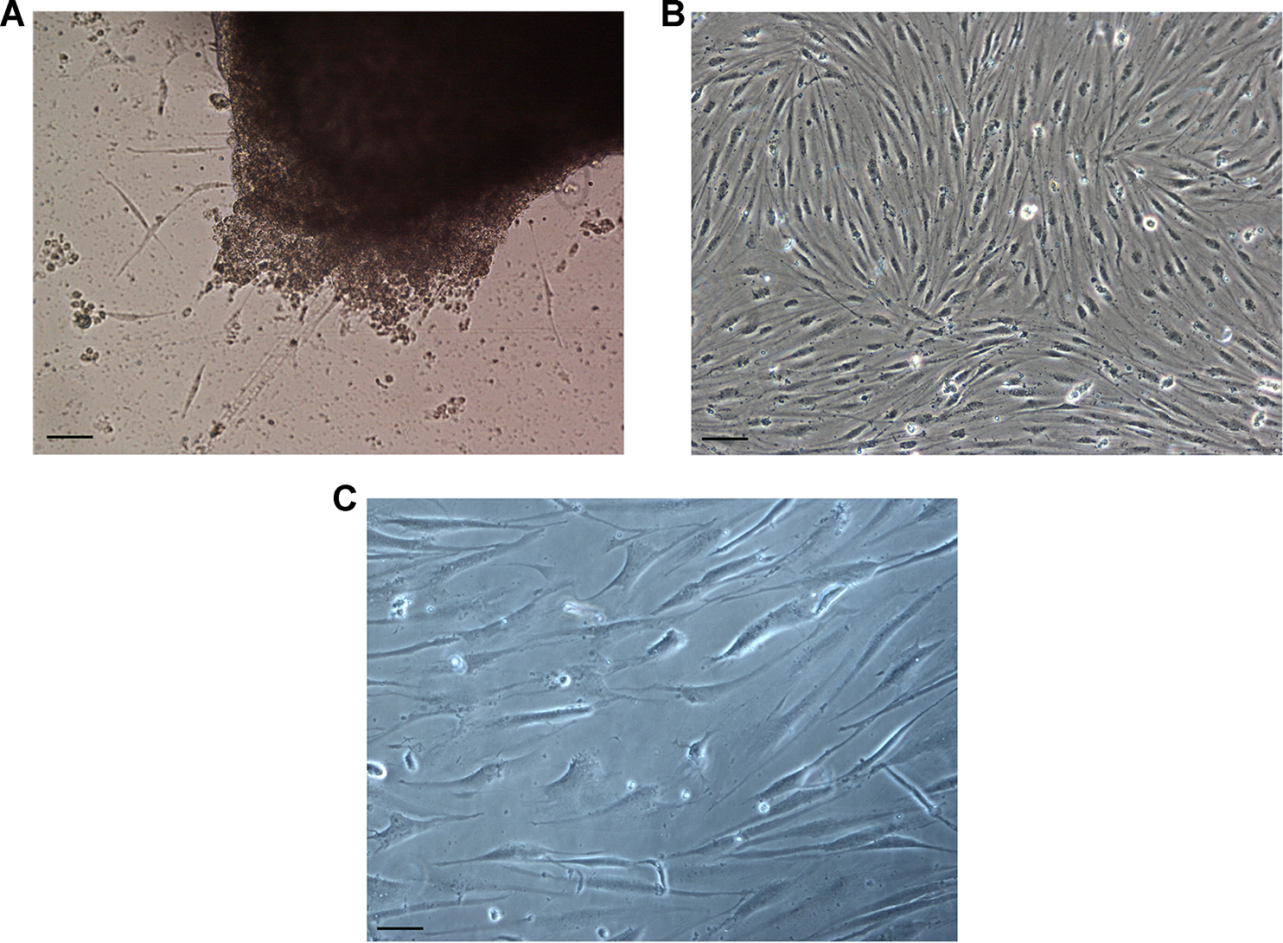
**Development of the *Anguilla* EL (eel liver) cell line**. (A) Cell culture and cell migration of the primary explants. (B) In vitro culture of EL cells at the 65th passage on the 5th day. (C) In vitro culture of the EL cells at the 40th passage on the 110th day (ECLIPSE TE2000‐S, Nikon, ×100, scale bars = 20 μm).

**Figure 2:**
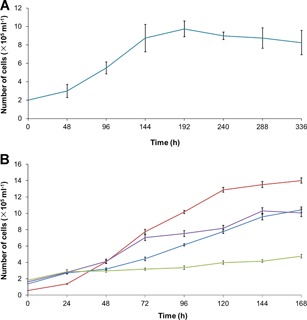
**The growth of the EL cell line at different passage numbers and temperature conditions**. (A) Growth curve of EL cells at the 28th passage (blue, 27°C). (B) Growth curve of EL cells at the 66th passage (green, 15°C; blue, 20°C; brown, 27°C; purple, 32°C). The maximum growth rate was obtained at 27°C, and the cell population doubling time (PDT) was significantly reduced, while the subculture continued. The values are displayed as mean ± standard error (SE) (*n* = 3).

Hepatic cells migrated outwards from several tissue explants on the third day following inoculation (Figure 1A). In the second week of incubation, radial outgrowths were observed, although no monolayer was formed in the primary culture for a period of 8 weeks. A confluent monolayer appeared at the fifth subculture, and the interval of the first subcultures varied from 30 to 40 days. The subcultures initially consisted of almost the same number of fibroblast‐like and epithelial‐like cells, as well as a limited number of stellate‐like cells. However, only fibroblast‐like cells were left after 10 subcultures. The multiplication interval was gradually reduced, following continuous subculture. This interval was estimated from 14 to 20 days between the 20th and 30th subculture and from 4 to 6 days after the 60th subculture (Figures 2A and 2B). The EL cell line was anchorage‐dependent, and resulted mainly in the formation of fibroblast‐like cells that were maintained in L‐15 containing 10% FBS (Figure 1B). The culture medium was changed every week and the EL cells were able to survive for 100 days or more in vitro (Figure 1C). From its initiation, this cell line was kept in in vitro culture over 72 months continuously, and was subcultured for more than 90 times. The 60th subculture of EL cells recovered from liquid nitrogen storage and could grow into a confluent culture in 2 days. The average viability of these cells was estimated from 75% to 85%.

### Growth studies

Among the three tested culture media, only L‐15 could support the growth of EL cells efficiently. When RPMI‐1640 medium was used the EL cells turned tumid and vacuolated, and they were not able to divide after subculture. In the presence of the Dulbecco's modified Eagle's medium, the EL cells changed into short fusiform‐like structures with pseudopodia and could not form a monolayer, while a massive mortality occurred during the second week following inoculation.

The EL cells were able to grow to a confluent monolayer at a temperature range between 15°C and 32°C, whereas at 10°C or 37°C, only small colonies were formed. The maximum growth rate was obtained at 27°C (Figures 2A and 2B). From 24 to 120 h following cell inoculation, the passage of 66 EL cells exhibited a logarithmic phase of growth with a PDT of 29.64 h, and thereafter a stagnate phase was noted (Figure 2B). In contrast to these observations, the passage 28 EL cells presented a logarithmic growth phase between 48 and 192 h after inoculation with a PDT of 84.68 h (Figure 2A).

### Chromosome analysis

**Figure 3:**
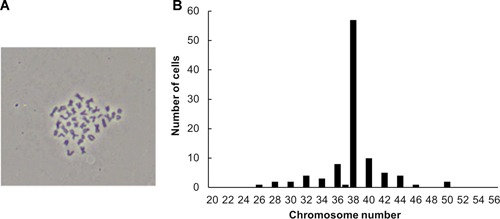
**Chromosome analysis of the EL cells at the 65th passage**. (A) Modal metaphase. (B) Chromosome number distribution. The main chromosome number was 38, which consisted of six pairs of mediocentrics, three pairs of subtelocentrics, and 10 pairs of telocentrics (2*n* = 6 m + 3st + 10t).

A total of 100 metaphase samples were obtained at the 65th passage and counted. The modal diploid karyotype of 2*n* = 38 (Figure 3A) was present in 57% of these samples, of which the metacentric, submetacentric, and telocentric chromosomes were those that corresponded to the chromosome pairs of 6, 3, and 10, respectively (2*n* = 6 m + 3sm + 10t). Chromosome analysis indicated a distribution of the chromosome number of the EL cell line. This number varied from 26 to 50 (Figure 3B).

### 18S rRNA sequence analysis

The origin of the EL cell line was confirmed by the 18S rRNA gene analysis. An expected PCR product of 1,714 bp was obtained using specific amplification of 18S rRNA from the extracted total genomic DNA. This was proven 100% identical to the published *A. anguilla* 18s rRNA sequence (GenBank: FM946070.1). The homologous analysis further indicated a complete match with *A. anguilla*.

### Susceptibility test

**Figure 4:**
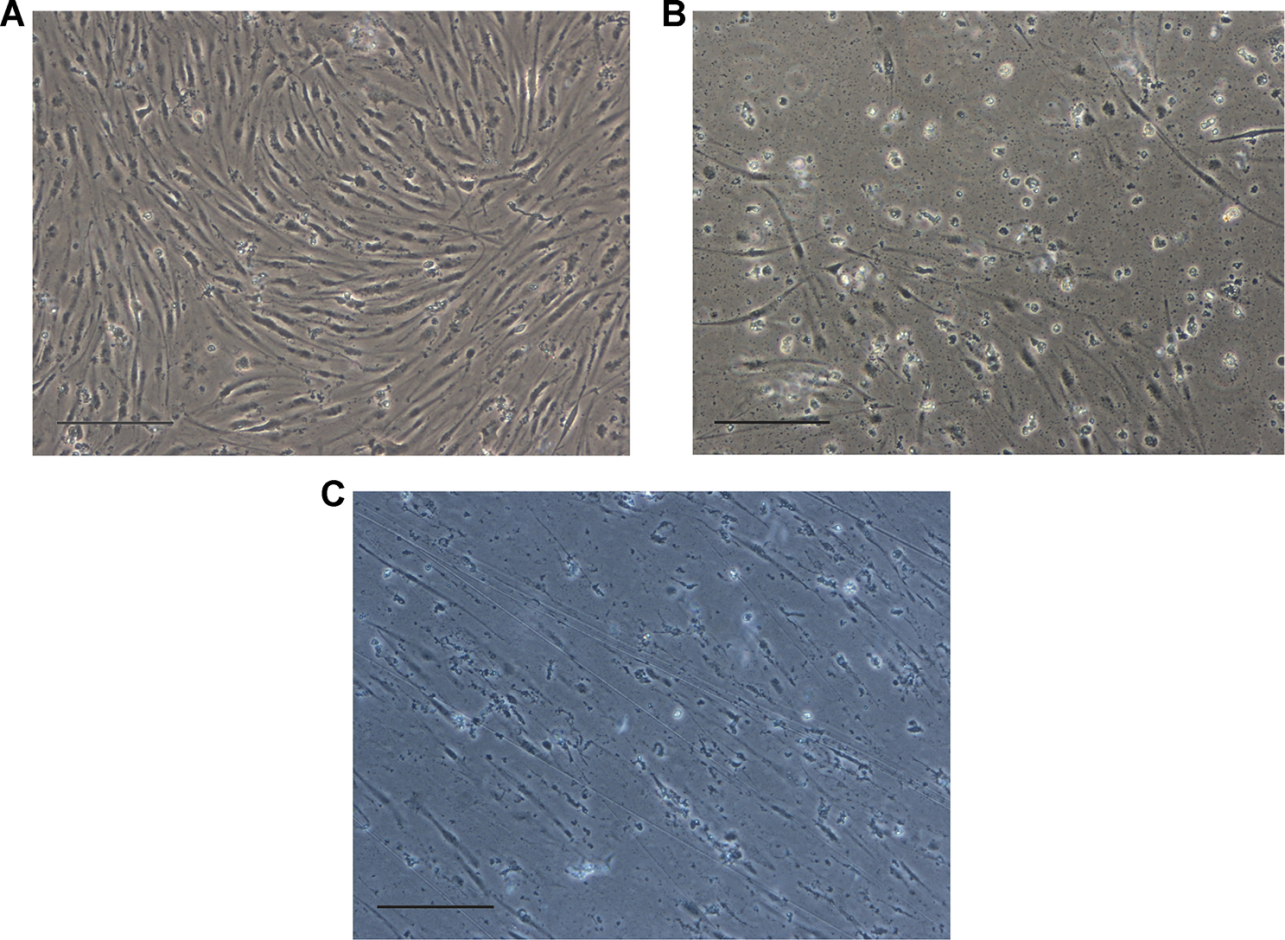
***Herpesvirus anguillae* (HVA) and *Rana grylio* virus (RGV) infection on EL cells at the 60th passage**. (A) Control cells following 168 h of culture. (B) EL cells at the 168 h time period following HVA inoculation. (C) EL cells following 120 h of culture and RGV inoculation. Multiplicity of infection (MOI) = 4.0 (ECLIPSE TE2000‐S, Nikon, ×100, scale bars = 50 μm).

**Figure 5:**
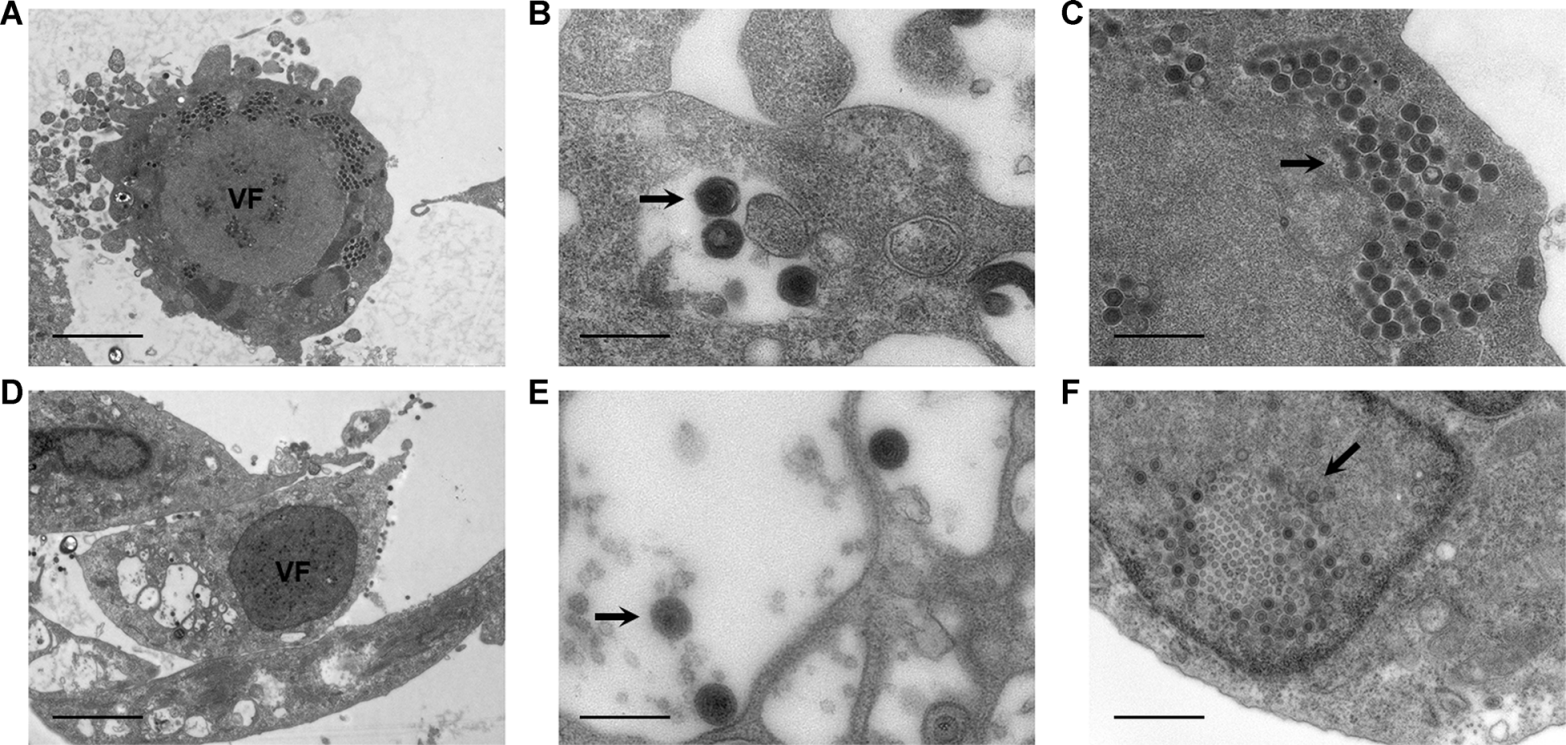
**Electron microscopy of the EL cells infected by *Herpesvirus anguillae* (HVA) and *Rana grylio virus* (RGV)**. (A and D) Expanded viral factory (TECNAI G2 20 TWIN, FEI, ×2,000, scale bars = 5 μm). (B and E) Arrows indicate the viral particles scattered in the cytoplasm (TECNAI G2 20 TWIN, FEI, ×15,000, scale bars = 1 μm) (C and F) the viral inclusion bodies with crystalline structures (TECNAI G2 20 TWIN, FEI, ×8,000, scale bars = 1 μm).

Both of the two tested viruses were able to cause cytopathic effect (CPE) on the EL cells. At 72 h after viral infection, the cells that were infected with HVA exhibited increased mortality. The mortality rate peaked at 168 h following viral infection with a mass cell shrinkage and exfoliation (Figure 4B). At 240 h following viral infection, the mortality rate was higher than 95%. The cell death was initially observed at 48 h after viral infection in the RGV‐inoculated group and the virus covered over 95% of the samples at 120 h following infection (Figure 4C) compared with the control cells that remained healthy (Figure 4A). The particles of both viruses were found in the cytoplasm and the nuclei and were determined by electron microscopy. Expanded viral factories (VF) were noted in the RGV‐infected cells, in which electron density was lower and/or higher than that of the surrounding cytoplasm (Figure 5A) (Zhang and Gui, 2012). In contrast to RGV‐infected cells, the VFs in HVA‐infected cells were localized in a vast area of the nuclei (Figure 5D). Viral particles were also found dispersed in the cytoplasm in both groups (Figures 5B and 5E). Multiple crystalline‐arranged RGV particles were found nearby the VF (Figure 5C), and numerous empty capsids and incomplete viral particles were noted within the HVA inclusions (Figure 5F).

Following PCR amplification of the *HVA* DNA polymerase gene, the expected 390 and 622 bp products were readily detected (Figure S1) compared with the published *HVApol* gene (van Beurden et al., 2010). The qRT‐PCR standard curve for RGV main capsid protein was plotted using linear‐regression analysis according to the sequencing report of the pMD‐19T‐MCP vector: *y* = −3.2966x + 36.508, *R* ^2^ = 0.9982, 2 ≤ *x* ≤ 8. The transcripts of MCP increased significantly in the EL cells from 6 to 72 h following infection with RGV (Figure S2).

### Induction of immune‐related gene expression

**Figure 6:**
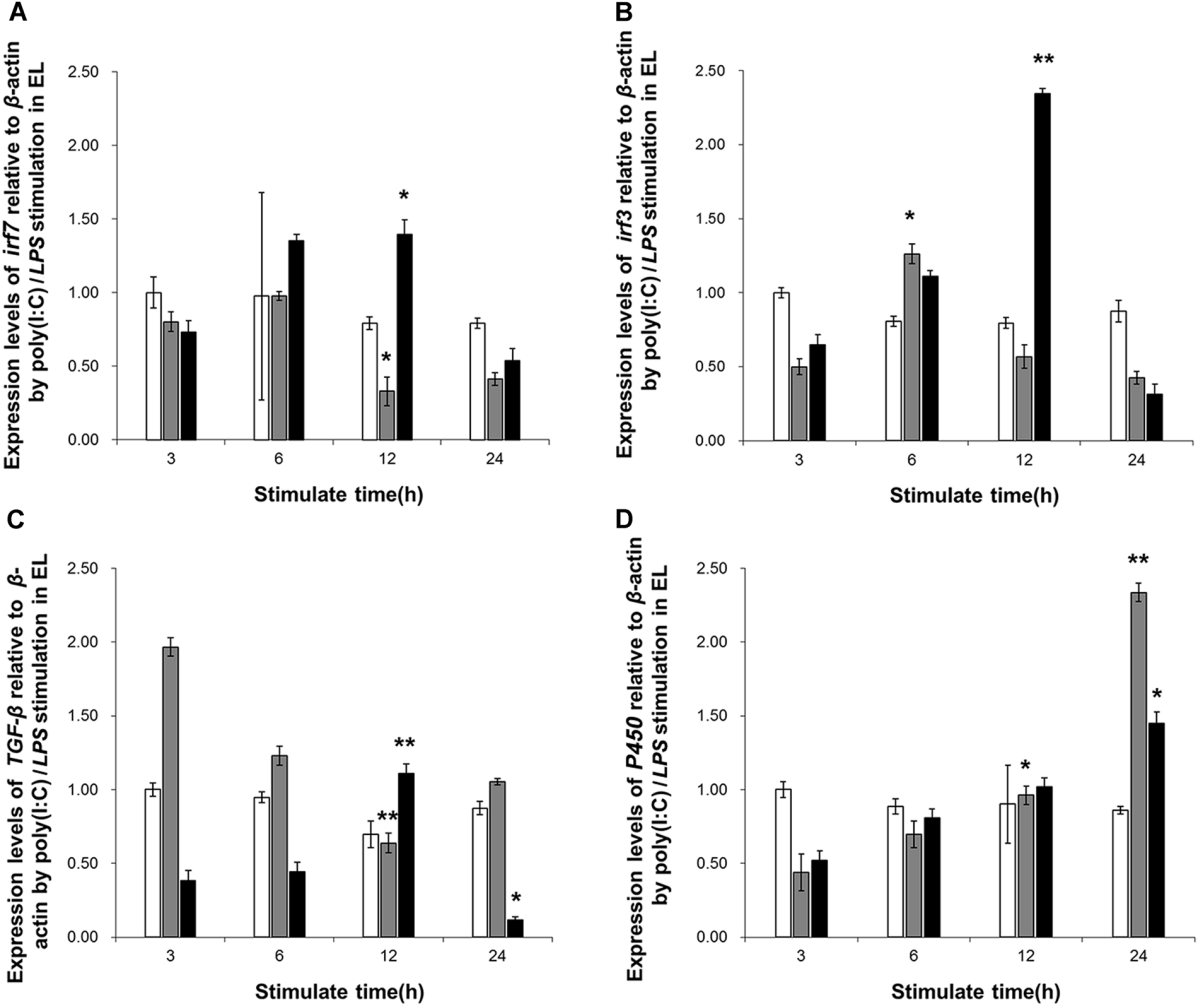
**Quantitative real‐time polymerase chain reaction (qRT‐PCR) of messenger RNA (mRNA) expression levels of the immune‐related genes in EL cells following immune stimulation**. Analysis of the mRNA expression levels of (A) interferon regulatory factor‐7 (*irf7*), (B) interferon regulatory factor‐3 (*irf3*), (C) transforming growth factor‐*β*1 (*TGF*‐*β*1), and (D) cytochrome *P450* in EL cells following poly(I:C)(gray) or lipopolysaccharide (LPS) (black) induction. The untreated group (white) is also shown, and *β*‐actin was used as an internal control. Each experiment was repeated at least three times. The values are displayed as mean ± standard error (SE) (*P < 0.05; **P < 0.01; *n* = 3).

In the poly (I:C) induction test, the expression levels of *irf7* and *TGF‐β1* were significantly downregulated. The maximum decrease was estimated to 58.75% and 67.51%, respectively, at 12 h post‐stimulation *(P* < 0.01) (Figures 6A and 6C). In contrast to these two genes, the expression of *irf3* and *CYP450* was upregulated. The expression levels of the *irf3* transcripts reached the peak at 6 h post‐stimulation with an increase of 2.52‐fold (*P *< 0.05) and subsequently returned to the initial levels (Figure 6B). The expression levels of *CYP450* increased gradually and exhibited a significant difference at 12 h post‐stimulation, while the levels peaked at 24 h post‐stimulation with an increase in the ratio of 5.32‐fold (*P* < 0.01) (Figure 6D).

In the LPS challenge test, the expression levels of all the four tested genes were upregulated, while the levels of *irf3, irf7*, and *TGF‐β1* decreased within 24 h. The expression levels of *irf7* indicated a significant increase at 6 and 12 h following stimulation, with a maximum ratio of 1.92‐fold (Figure 6A) (12 h, *P* *<* 0.01). The expression levels of *irf7* returned subsequently to the initial levels (Figure 6A). The expression levels of *irf3* were significantly upregulated from 6 to 12 h and the highest ratio was estimated to 3.62‐fold (12 h, *P* < 0.01). *Irf3* expression levels were downregulated with a 52% decrease (24 h, *P* < 0.01) (Figure 6B). The expression levels of *TGF‐β1* peaked at 12 h (2.92‐fold increase, *P* < 0.01) and were slightly reduced at 24 h (68% decrease, *P* < 0.01) (Figure 6C). In addition, the expression levels of *CYP450* were upregulated continuously at 6 h post‐stimulation with a maximum increase of 2.79‐fold (24 h, *P* < 0.01) (Figure 6D). The expression of the four genes in the untreated group did not show any significant changes in both induction tests.

## Discussion

The EL cell line required a considerably large time period for its primary culture and early subcultures. The cells indicated prolonged multiplication intervals on more than 50 passages compared with those noted in other reported marine fish cell lines (Fryer and Lannan, 1994; Lakra et al., 2011). These findings could be attributed to the origin of the experimental animals used. As a European‐origin catadromous teleost (Schmidt, 1923), the artificial reproduction of *A. anguilla* is a difficult task. Since the wild‐type embryo and the neonatal leptocephalus are mainly present in the oceans, the glass eels are the predominant catches collected in the European coastal waters. However, their export was restricted after being considered endangered species in 2009. Therefore, the elvers are the youngest eel to be collected from the fish farmers in China. The 20 g elvers had been shown to be at their growth peak in the ponds (Huang and Cheng, 1997) and their tissues and cells were already highly differentiated. Furthermore, the several types of parasites discovered in eel viscera and muscles may also negatively affect the growth potential of early in vitro cultures.

The karyotype analysis revealed that 57% of the passage 65 EL cells presented a diploid chromosome number of 2*n* = 38, which was identical with the model number of *A. anguilla* (Yang et al., 1999). Although the EL strain did not exhibit the ability to form neoplasms in vitro, its aneuploidy implied the possibility of immortality (Chen et al., 2005; Bryson et al., 2006). The EL cells were not sensitive to slight osmolality changes, which may be due to the euryhalinity of *A. anguilla* (Schmidt, 1923).

In the present study, the cell line sample that exhibited the longest longevity was retained for over 6 months at 20°C. The cell lines that are maintained in vitro for a long time period exhibit mutations that may alter their original functional characteristics that have been identified at earlier passage levels (Yu et al., 1997). Their behavior modes can also be altered (Hughes et al., 2007). Two massive cell deaths were noted at the 3rd, 48th, 49th, and 50th subculture, which suggested the optimal time periods for the intrinsic in vitro transformation of the EL cell line (Doyle and Griffiths, 2000). Long‐term subculture could also place selective pressure on cell proliferation, which may explain the disappearance of the epithelial‐like and stellate‐like cells during subcultivation.

Nearly, all fish cell lines are anchorage‐dependent and a large number of primary cultures have been prepared from fish liver compared with other types of solid fish tissues (Baksi and Frazier, 1990). A surprising feature noted in certain species was the inability of hepatocytes to attach and spread using growth‐related substrates, which suggested that hepatic cell lines could release attachment and spreading factors that promote the preparation of primary hepatocyte cultures (Bols and Lee, 1991). Similar phenomena were also observed during the early development of the EL cell line. The cell line required considerably higher inoculum density during adherence to a glabrous inner surface of the culture flask, while cells with lower density remained at the bottom and could grow into a confluent monolayer following removal of the supernatant. Therefore, the asymmetrical subculture method was selected.

The susceptibility of the EL cell line to HVA and RGV infection was demonstrated in the present study. The infection course required a considerable time period in EL cells, indicating the potential for HVA‐prevention studies compared with former HVA tests using the EO cell line (Ge et al., 2014) Iridoviruses are common pathogens detected in *A. japonica* (Sorimachi and Egusa, 1987) and other aquaculture species in the Fujian province (Yang et al., 1999). The present study indicated that the EL cells could be used as a universal model for viral isolation.

The rapid response kinetics of the gene expression levels of various immune factors with regard to in vitro or in vivo inflammatory stimulation has been confirmed repeatedly in teleosts (Sudhakumari et al., 2005; Haddad et al., 2008; Maehr et al., 2013; Huang et al., 2014). However, the mechanism of inflammatory‐induced immune modulation remains ambiguous. Recently, cell lines have been used as tools for functional analysis of fish innate immunity genes. The *IRF* family members play critical roles in the cellular differentiation of hematopoietic cells, in the regulation of gene expression in response to pathogen‐derived danger signals, and consequently in the regulation of the cell cycle and apoptosis (Tamura et al., 2008). *Irf3* and *irf7* are highly expressed in the teleost liver (Yao et al., 2012). *Irf3* has been reported to be upregulated in both peripheral blood leukocytes and in vivo models following poly (I:C) or *LPS* stimulation in *A. anguilla*. This was confirmed in the present study, by the alterations of the *irf7* levels following *LPS* induction. It is interesting to note that the expression levels of *irf7* were downregulated in response to poly (I:C) in the present study, compared with a previous in vivo study (Huang et al., 2014). This may be due to the absence of some protein members that participate in multiple cross‐talk in vivo signaling pathways.

The *CYP* gene superfamily members are usually involved in the detoxification of exogenous chemicals and the metabolism of various endogenous substrates (Uno et al., 2012). Since the 1980s, hepatic expression of *CYP*s in *Oncorhynchus mykiss* (Walbaum 1792) and other fishes has been considered a common biomarker to assess contamination of the aquatic environment (Winston et al., 1988; Nilsen et al., 1998; Bugiak and Weber, 2009). In the present study, the expression levels of *CYP450* indicated a significant long‐term upregulation following poly (I:C) and/or *LPS* exposure, indicating that the EL cell line could retain the intrinsic characteristics of its hepatic origin. This renders the EL cell line an ideal model for toxicological and pharmacological analysis of aquatic pollutants.

## Conclusion

In summary, the present study demonstrated that EL is a diploid cell line of *A. anguilla* that has been established from its hepatic tissues. This cell line can be potentially used for the studies of infectious viral diseases of Anguillidae, and for the studies that investigate the protection of this endangered species.

## Conflict of interest

All authors declare that they have no any conflicts of interest.

## Supporting information

Supporting information.

Supporting information.

Supporting information.
