# Supplementary figures and images for: Development and characterization of a continuous cell line (EL) from the liver of European eel Anguilla anguilla
Source: Cell Biol Int. 2019 Dec 19;44(3):808–20. doi: 10.1002/cbin.11276 (PMC7028054; doi:10.1002/cbin.11276)

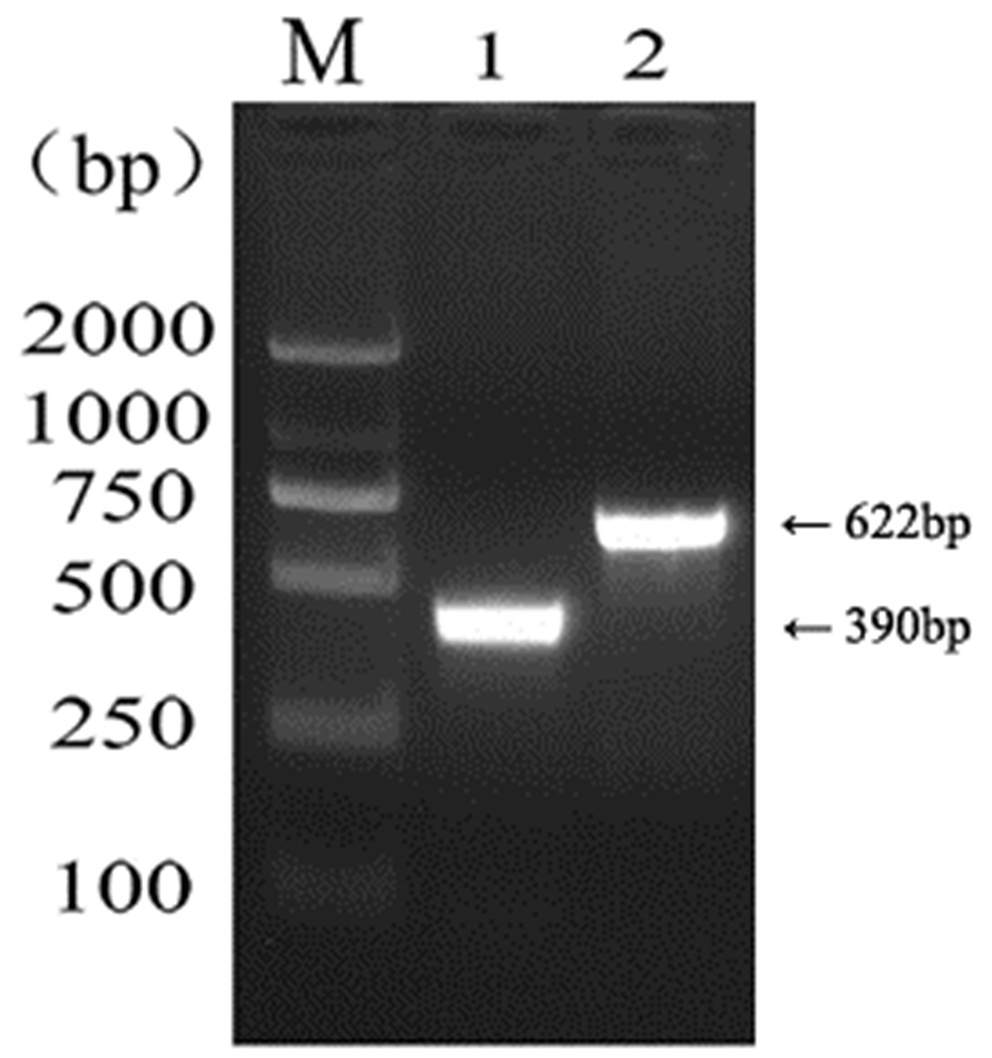

Supplement: Supplementary file 1 — Supporting information. [file CBIN-44-808-s001.tif]

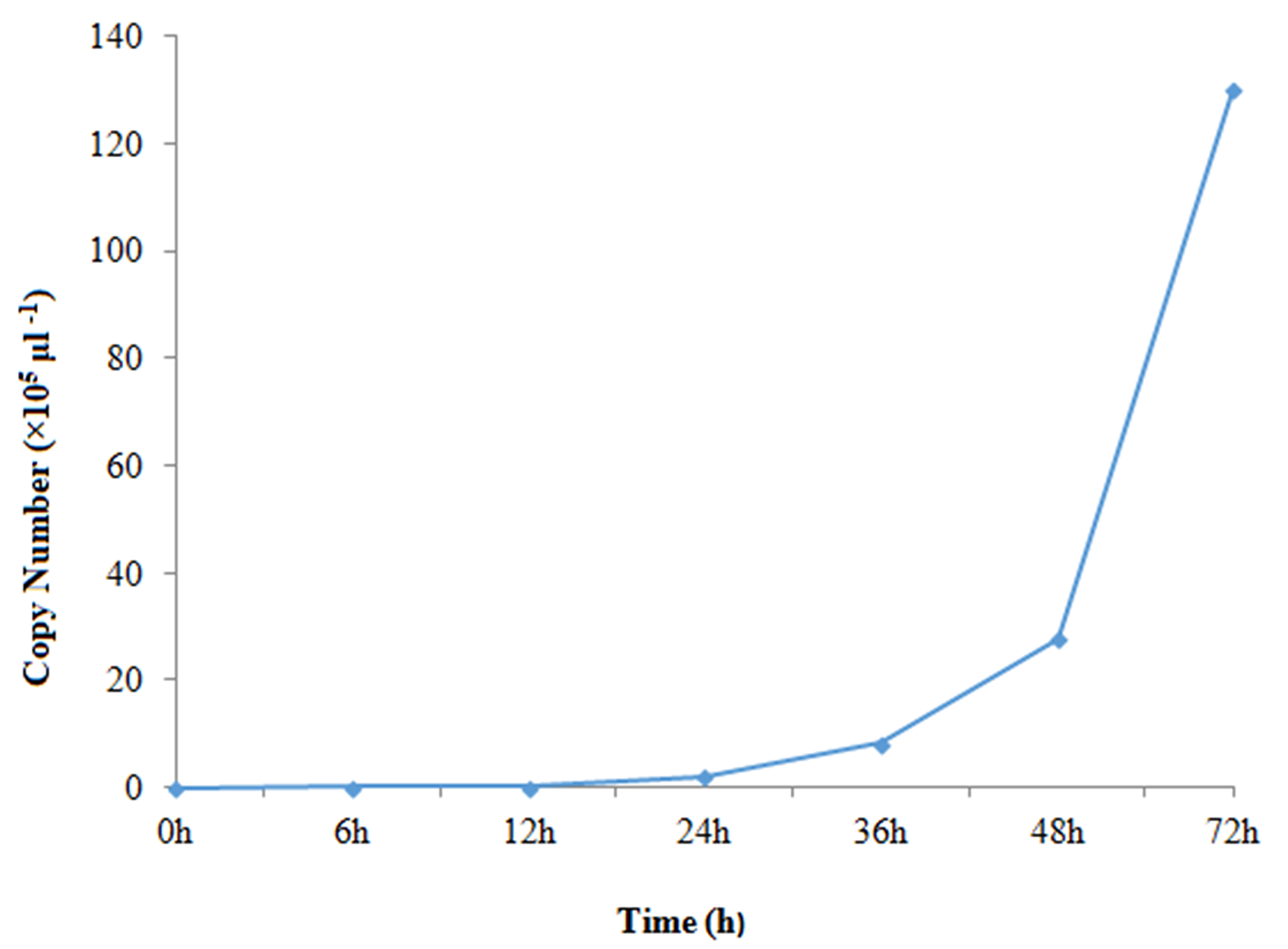

Supplement: Supplementary file 2 — Supporting information. [file CBIN-44-808-s002.tif]
