# Supplementary material for: Development and characterization of a continuous cell line (EL) from the liver of European eel Anguilla anguilla
Source: Cell Biol Int. 2019 Dec 19;44(3):808–20. doi: 10.1002/cbin.11276 (PMC7028054; doi:10.1002/cbin.11276)
Supplement: Supplementary file 3 — Supporting information. [file CBIN-44-808-s003.docx]

**Figure S1: Agarose gel electrophoretic pattern of the PCR products corresponding to the expression of the *Herpesvirus anguillae* DNA polymerase gene in the passage 62 EL cells following infection.**

Lane M, DL 2000 DNA marker; lane 1, *HVApolF/polR* products; lane 2, *HVAF/HVAR* products. Following PCR amplification of the HVA DNA polymerase gene, the expected 390 and 622 bp products were readily detected compared with the products corresponding to the published *HVApol* gene.

**Figure S2: RGV major capsid protein expression in EL cells following infection.**

The qRT-PCR standard curve for the expression of the RGV *main capsid protein* (*MCP*) gene was plotted using linear-regression analysis according to the sequencing report of the pMD-19T-MCP vector. The following formula was used: y = -3.2966x + 36.508; R^2^ = 0.9982, 2≤x≤8. The transcripts of *MCP* increased significantly in the EL cells from 6 to 72 h following infection with RGV.
